# Hybridization and introgression of the mitochondrial genome between the two species *Anisakis pegreffii* and *A. simplex* (s.s.) using a wide genotyping approach: evolutionary and ecological implications

**DOI:** 10.1017/S0031182025000228

**Published:** 2025-03

**Authors:** Simonetta Mattiucci, Marialetizia Palomba, Beatrice Belli, Renato Aco-Alburqueque, Paolo Cipriani, Xavier Roca-Gerones, Mario Santoro, Stephen C. Webb, Giuseppe Nascetti

**Affiliations:** 1 Department of Public Health and Infectious Diseases, Section of Parasitology, Sapienza University of Rome, Rome, Italy; 2 Department of Ecological and Biological Sciences (DEB), Tuscia University, Viterbo, Italy; 3 Department of Integrative Marine Ecology, Stazione Zoologica Anton Dohrn, Naples, Italy; 4 Institute of Marine Research (IMR), Nordnes, Bergen, Norway; 5 Secció de Parasitologia, Departament de Biologia, Sanitat i Medi Ambient, Facultat de Farmàcia i Ciències de l’Alimentació, Universitat de Barcelona, Barcelona, Spain; 6 Private Bag 2, Nelson 7042, Cawthron Institute, Nelson, New Zealand

**Keywords:** *Anisakis*, DNA microsatellites, hybridization, mitochondrial introgression, mtDNA *cox*2, SNPs

## Abstract

*Anisakis pegreffii* and *A. simplex* (s.s.) are the two zoonotic anisakids infecting cetaceans as well as pelagic/demersal fish and squids. In European waters, *A. pegreffii* prevails in the Mediterranean Sea, while *A. simplex* (s.s.) in the NE Atlantic Ocean. Abiotic conditions likely play a significant role in shaping their geographical distribution. The Iberian Atlantic and Alboran Sea waters are sympatric areas of the two species. A total of 429 adults and L3 stage from both sympatric and allopatric areas were studied by a wide nuclear genotyping approach (including newly and previously found diagnostic single nucleotide polymorphisms (SNPs) at nuclear DNA (nDNA) and microsatellite DNA loci) and sequenced at mitochondrial DNA (mtDNA) *cox*2. Admixture between the two species was detected in the sympatric areas studied by STRUCTURE Bayesian analysis; NEWHYBRIDS revealed different categories of hybridization between the two species, representing approximately 5%. A tendency for F1 female hybrids to interbreed with the parental species at the geographical distribution limits of both species was observed. This finding suggests that hybridization occurs when the two parental species significantly differ in abundance. Mitochondrial introgression of *A. simplex* (s.s.) in *A. pegreffii* from Mediterranean waters was also detected, likely as a result of past and/or paleo-introgression events. The high level of genetic differentiation between the two species and their backcrosses indicates that, despite current hybridization, reproductive isolation which maintains evolutionary boundaries between the two species, exists. Possible causes of hybridization phenomena are attempted, as well as their evolutionary and ecological implications, also considering a sea warming scenario in European waters.

## Graphical abstract

**Figure:**
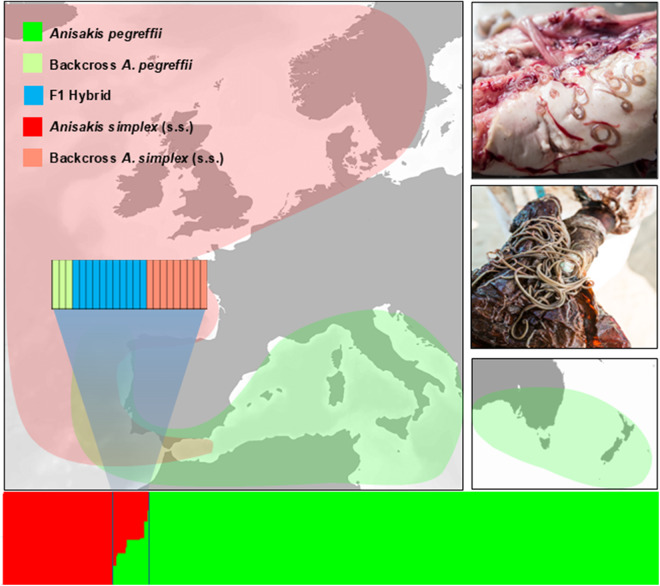

## Introduction

The evolutionary boundary between closely related parasite species represents an important window to study microevolutionary processes, speciation mechanisms, genetic architecture and gene flow between interacting species. Such boundaries are often permeable, leading to natural hybridization phenomena in contact zones. Currently, attention is directed towards describing patterns of genetic variation across the genome of closely related parasites species to disentangle and characterize patterns of ongoing hybridization and introgression phenomena (Calvani and Slapeta, 2020).

Previous genetic, phylogenetic, morphological and ecological evidence supports the view that the two anisakid species of the *Anisakis simplex* (s.l.) complex, i.e. *A. pegreffii* and *A. simplex* (s.s.), constitute two well-genetically differentiated sister taxa (Mattiucci et al., 2014). The two species differ in geographical distribution, phenotypic traits – such as differential *intra-vitam* (reviewed in Mattiucci et al., 2018) and *post-mortem* migration capability in the musculature of their intermediate/paratenic fish hosts (Cipriani et al., 2016, 2024) – as well as differential gene expression of some adaptive and immunomodulatory molecules under thermal cues (Palomba et al., 2019, 2020a). Certain environmental abiotic conditions, such as sea temperature (Gomes et al., 2023; Mladineo et al., 2023), represent determinant drivers in shaping their geographical ranges (Mattiucci et al., 2018; Diez et al., 2022; Díez et al., 2024). Similarly, biotic variables, such as host availability, diversity and preference, also play a significant role (Mattiucci and Nascetti, 2008; Klimpel et al., 2010; Kuhn et al., 2011; Shamsi and Barton, 2023). Indeed, both species share a wide range of definitive hosts, mostly oceanic dolphins, mysticetes and phocoenids (Mattiucci et al., 2018; Cipriani et al., 2022). The range of their intermediate/paratenic hosts includes a wide array of demersal and pelagic fish and squids, within the geographical distribution of the two species (reviewed in Mattiucci et al., 2018; Shamsi and Barton, 2023). The two species exhibit allopatric zonation following a latitudinal gradient. *Anisakis pegreffii* is widespread in the Mediterranean Sea and the Austral region, between 30°S and 60°S; in the Atlantic, its northern distribution limit is the Iberian coast up to the Bay of Biscay (Diez et al., 2022; Díez et al., 2024). *A. pegreffii* is also widely distributed in the South Pacific Ocean, from the East China Sea (Lee et al., 2009; Ding et al., 2022) to New Zealand waters, in sympatric condition with *A. berlandi* (Mattiucci et al., 2018; Bello et al., 2021; Shamsi and Barton, 2023). Genetic sub-structuring of *A. pegreffii* from Austral and Boreal regions, as inferred from population genetics analyses using microsatellite loci (Mattiucci et al., 2019), suggests that geographic distance restricts gene flow between ‘antipodean’ populations of this parasite species. Despite the genetic sub-structuring, genetic differentiation (*Fst*) values observed at 5 simple sequence repeat (SSR) loci indicate a high gene flow (*Nm* = 7.8) between Boreal and Austral populations of *A. pegreffii* (Mattiucci et al., 2019). This genetic pattern is likely shaped by different cetacean populations, oceanographic barriers and the historical microevolutionary lineage diversification of the species in distinct waters (Mattiucci et al., 2014, 2019, 2025; Blažeković et al., 2015; Ding et al., 2022).

*Anisakis simplex* (s.s.) distribution ranges approximately from 35°N to the Arctic Seas, circumpolarly. Its southern geographic limit in North-East (NE) Atlantic waters seems to be the Iberian Atlantic coast (see Mattiucci et al., 2018). However, in some regions, the geographic ranges of the two *Anisakis* species (i.e. *A. pegreffii* and *A. simplex* (s.s.)) overlap. These regions include the NE Atlantic Ocean (Spanish−Portuguese coast) (Abollo et al., 2001; Pontes et al., 2005; Mattiucci et al., 2016; Cipriani et al., 2022; Ramilo et al., 2023) and the Western Pacific Ocean (the East China Sea and the Sea of Japan), (Lee et al., 2009; Suzuki et al., 2010; Chou et al., 2011; Quiazon et al., 2011; Ding et al., 2022), where they are often found in sympatry and syntopy, at both larval and adult stages.

In these contact areas of their geographical distribution, the occurrence of ‘putative hybrids’ or ‘recombinant genotypes’ between *A. pegreffii* and *A. simplex* (s.s.) has been reported, either as larval stages or as adults (Abollo et al., 2003; Martín-Sánchez et al., 2005; Umehara et al., 2006; Lee et al., 2009; Suzuki et al., 2010; Chou et al., 2011; Quiazon et al., 2011; Molina-Fernández et al., 2015; Abattouy et al., 2016; Gómez-Mateos et al., 2020), as well as from allopatric regions such as the Mediterranean Sea (Farjallah et al., 2008; Meloni et al., 2011; Cavallero et al., 2012, 2014; Chaligiannis et al., 2012; Pekmezci et al., 2014). However, these studies were typically based on a single marker (i.e. the internal transcribed spacer (ITS) region of rDNA), lacking the resolution to determine whether the observed shared polymorphism between the two taxa was due to incomplete lineage sorting, historical introgression, ongoing hybridization and/or introgression. Indeed, hybridization, introgression and the retention of ancestral polymorphism in closely related parasitic taxa could generate patterns of genetic variation that complicate their disclosure, when choosing an unsuitable number of genetic/molecular markers (Anderson, 2001). Mattiucci et al. (2016) used a multilocus approach with nuclear markers (allozyme loci and *EF1 α* nuclear DNA (nDNA) sequence analysis) to distinguish parental taxa and F1 hybrids among *A. pegreffii* and *A. simplex* (s.s.) specimens sampled along the Iberian Atlantic coast. Similarly, Roca-Geronès et al. (2021) detected F1 hybrids in the same sympatric area using the same markers. Both studies, however, reported discordant results when applying polymerase chain reaction–restriction fragment length polymorphism (PCR–RFLP) analysis of the ITS region of rDNA to the same specimens analysed with the multilocus approach. This highlights the risk of misidentifying parental *Anisakis* species and their hybrid categories (F1 hybrids and backcrosses) when relying solely on a single nuclear marker. Given that a large number of diagnostic markers are required to accurately identify F1 and Fn hybrid categories (i.e. backcrosses) between closely related species (Tarroso et al., 2014), a multilocus approach is essential for characterizing hybrid gene pools. True hybrid events in contact zones between closely related species involve amalgamations of independently evolved genomes, leading to intergenomic interactions. In these cases, evolutionary processes such as recombination and selection can occur, making it essential to study these phenomena using joint data from both nuclear and cytoplasmic markers. In this context, the combined use of some DNA microsatellite loci developed in species of the *A. simplex* (s.l.) complex (Mattiucci et al., 2019; Bello et al., 2020) and *Nas10* nDNA (Palomba et al., 2020b) has allowed to describe the first report on the natural interspecific hybridization between *A. pegreffii* and *A. berlandi* in one of their contact areas (Bello et al., 2021) included in the species’ geographical ranges of distribution.

The use of microsatellite DNA loci has revealed the existence of sex-linked loci in the three species of the *A. simplex* (s.l.) complex (Mattiucci et al., 2019; Bello et al., 2021). These are genes located on the X chromosome. These chromosomes are maternally inherited, as they lack a paternal chromosomal counterpart and thus possess only a single copy of the gene. In general, in ascaridoid nematodes, males are XO, and females are XX (Müller and Tobler, 2000; Foster et al., 2020). However, exceptions are common, such as in *Ascaris suum*, which possesses 19 autosomes and 5 X chromosomes (2n = 38A + 10X in females, 38A + 5X in males) (Müller and Tobler, 2000). Preliminary cytogenetic studies recently conducted *in vitro* on *A. pegreffii* have shown that this parasite species has a diploid chromosome number of 2n = 18A (Moratal et al., 2023). However, no distinct chromosomal organization patterns between males and females were reported.

In the present paper, a multilocus genotyping approach was applied on several specimens of *A. pegreffii* and *A. simplex* (s.s.), incorporating a broader set of novel and previously detected diagnostic nuclear and mitochondrial loci. Additionally, a Bayesian population structure approach was applied and validated for the two species. The aims were to: (1) develop and validate new diagnostic nuclear markers between the two species; (2) define the genetic structure of the two species in the contact zone spanning the Atlantic Iberian coast and Mediterranean waters of the Alboran Sea, in comparison with their allopatric populations; (3) assess the occurrence of different patterns of ongoing hybridization between the two interacting species in this sympatric area; (4) detect mitochondrial introgression in their genome; (5) investigate the causes of the distribution of these hybridization/introgression phenomena, as well the potential evolutionary and ecological implications of the findings; and, finally, (6) disclose the potential occurrence of sex-linkage in the newly scored SSR loci.

## Materials and methods

### Sample collection

**Figure 1.**
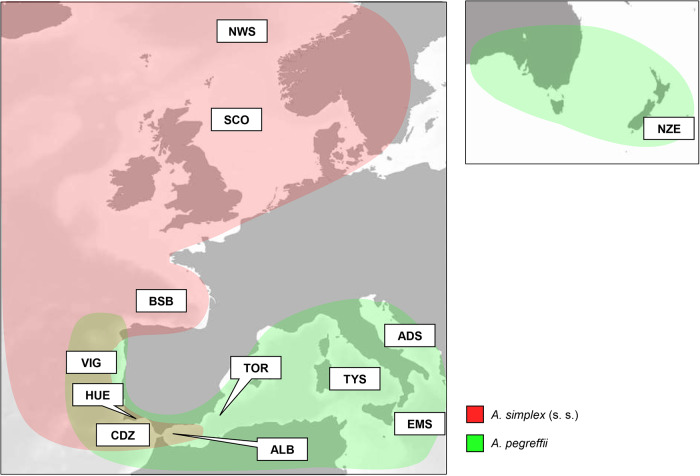
Collecting sites of *A. simplex* (s.s.) and *A. pegreffii* analysed in the present study, mapped onto the geographical range of the two parasite species (coloured in red and green, respectively). ADS, Adriatic Sea; TYR, Tyrrhenian Sea; IOS, Ionian Sea; TOR, Torrevieja (Mediterranean Spain coast); ALB, Alboran Sea; CDZ, Cadíz (Atlantic Spain Coast); HUE, Huelva (Atlantic Spain Coast); VIG, Vigo (Atlantic Spain Coast); BSB, Bay of Biscay; SCO, Seas West of Scotland; NWS, Norwegian Sea; NZE, New Zealand coast.

A total of N = 429 *Anisakis* spp. specimens were collected from sampling localities in the NE Atlantic Ocean, Mediterranean Sea and South Pacific waters (New Zealand) (Fig. 1). These locations include regions where the two sibling species *A. simplex* (s.s.) and *A. pegreffii* are reported both in allopatry (e.g. the North Sea for *A. simplex* (s.s.); the Central-Eastern Mediterranean Sea and New Zealand waters for *A. pegreffii*) and in sympatry (e.g. the Atlantic Iberian Peninsula and the Alboran Sea). Specifically, N = 314 third-stage larvae (L3) were collected from various fish hosts, while N = 115 adult nematodes were obtained from cetacean host species. The adult specimens were first sex-discriminated according to the main morphological diagnostic features between males and females (Mattiucci et al., 2018). Details about sample collections, including the localities of the intermediate/paratenic (fish) and definitive (cetacean) hosts and the number of adult male and female specimens studied, are reported in Table 1. Collected nematodes were repeatedly washed in saline solution and preserved by freezing at −20 °C in distilled water or stored in 70% alcohol until their genotyping.

**Table 1.** Number of specimens analysed from definitive and intermediate/paratenic hosts reported with sampling area, genotyped at nuclear (*EF1 α*-1, *Nas10, Sod* and *Adk* nDNA and SSR loci) and mitochondrial (mtDNA *cox*2) gene loci

Sampling area	Code area	Host species	N	N_A♂♂_	N_A♀♀_	N_L3_	N *cox*2 mtDNA	*EF1 α-1* nDNA	*Nas10* nDNA	*Sod* nDNA	*Adk* nDNA	SSR DNA loci
Mediterranean Sea												
Adriatic Sea (42°18ʹN − 15°35ʹE)	ADS	*Lophius piscatorius*	2	–	–	2	2	2	2	2	2	2
		*Merluccius merluccius*	2	–	–	2	2	2	2	2	2	2
		*Scomber scombrus*	3	–	–	3	3	3	3	3	3	3
		*Trachurus trachurus*	1	–	–	1	1	1	1	1	1	1
		*Stenella coeruleoalba*	18	3	15	–	18	18	18	18	18	18
Tyrrhenian Sea (41°7ʹN − 13°24ʹE)	TYS	*Lepidopus caudatus*	21	–	–	21	21	21	21	21	21	21
		*Merluccius merluccius*	2	–	–	2	2	2	2	2	2	2
		*Scomber scombrus*	4	–	–	4	4	4	4	4	4	4
Eastern Mediterranean Sea (39°93ʹN − 16°69ʹE)	EMS	*Merluccius merluccius*	102	–	–	102	102	102	102	102	102	102
		*Phycis blennoides*	20	–	–	20	20	20	20	20	20	20
Torrevieja (37°98ʹN − 0°30ʹW)	TOR	*Merluccius merluccius*	2	–	–	2	2	2	2	2	2	2
Alboran Sea (35°59ʹN − 3°41ʹW)	ALB	*Merluccius merluccius*	68	–	–	68	68	68	68	68	68	68
		*Trachurus trachurus*	3	–	–	3	3	3	3	3	3	3
NE Atlantic Ocean												
Gulf of Cádiz (36°19ʹN − 6°40ʹW)	CDZ	*Merluccius merluccius*	16	–	–	16	16	16	16	16	16	16
Huelva (36°50ʹN − 7°18ʹW)	HUE	*Merluccius merluccius*	18	–	–	18	18	18	18	18	18	18
Vigo (42°11ʹN − 9°45ʹW)	VIG	*Merluccius merluccius*	6	–	–	6	6	6	6	6	6	6
		*Delphinus delphis*	30	11	19	–	30	30	30	30	30	30
		*Globicephala melas*	3	–	3	–	3	3	3	3	3	3
		*Phocoena phocoena*	9	6	3	–	9	9	9	9	9	9
Bay of Biscay (44°13ʹN − 6°28ʹW)	BSB	*Micromesistius poutassou*	13	–	–	13	13	13	13	13	13	13
		*Trachurus trachurus*	1	–	–	1	1	1	1	1	1	1
Scottish Sea (59°13ʹN − 0°14ʹW)	SCO	*Lagenorhynchus albirostris*	5	–	–	5	5	5	5	5	5	5
		*Stenella coeruleoalba*	5	–	5	–	5	5	5	5	5	5
Norwegian Sea (68°52ʹN − 3°08ʹE)	NWS	*Clupea harengus*	13	–	–	13	13	13	13	13	13	13
		*Micromesistius poutassou*	7	–	–	7	7	7	7	7	7	7
		*Scomber scombrus*	5	–	–	5	5	5	5	5	5	5
New Zealand (40°20’S − 172°37ʹE)	NZE	*Globicephala melas*	50	20	30	–	50	50	50	50	50	50
		Tot:	429	40	75	314	429	429	429	429	429	429

N_A ♂♂_, number of adult male nematodes; N_A ♀♀_, number of adult female nematodes; N_L3_, number of species at third larval stage.

### Multi-marker genotyping approach based on nuclear and mitochondrial genes

Approximately 2 mg of tissue was cut from each larval and adult specimen, using a portion from the cephalic end for adults. DNA extraction was performed using the Quick-gDNA Miniprep Kit (ZYMO RESEARCH), according to the manufacturer’s protocol. The 429 sampled individuals were first genotyped using nuclear markers (*EF1 α-1* and *Nas10* nDNA gene loci) previously developed for the *A. simplex* (s.l.) complex. PCR amplification of the elongation factor *EF1 α* nDNA was performed using the primers EF-F and EF-R (Table 2) following the procedures detailed in Mattiucci et al. (2016). For the *Nas10* nDNA gene, PCR amplification was performed using the primers *Nas10*-F and *Nas10*-R (Table 2) following the procedures detailed in Palomba et al. (2020b).

**Table 2.** Locus name, primers sequences and fragment bp size of the gene loci used in the present study. For SSR nuclear loci, the repeat type and the fluorescent dye used to label the primer are also reported

Locus	Primer sequence 5ʹ – 3’	Size (bp)			N_SNP_	References
mtDNA *cox*2	F: TTTTCTAGTTATATAGATTG	600				Nadler and Hudspeth 2000
	R: CACCAACTCTTAAAATTATC					
*EF1 α-1* nDNA	F: TCCTCAAGCGTTGTTATCTGTT	409			2	Mattiucci et al., 2016
	R: AGTTTTGCCACTAGCGGTTCC					
*Nas10* nDNA	F: GAGAGTCATTCTCGC	451			1	Palomba et al., 2020b
	R: TCATGTATTTCCGAC					
*Sod* nDNA	F: AGAAGGTGAGCCAACGACTG	706			6	present paper
	R: GGCATTTCCCGTCTTCAAGC					
*Adk* nDNA	F: AACAGGCCGTCTCTTCCATC	1062			8	present paper
	R: ACGAGGACTGCAGCTGAATC					
**SSR loci**		**Size range (bp)**	**Repeat type**	**Dye**	**N_A_**	
Multiplex 1						
*Anisl 05784*	F: GGGTTTGGACACTGGTTTGG*	69 – 105	(TGT) 11	VIC	16	Mattiucci et al., 2019
	R: TGCAATCGTCATTTCTGCCTC					
*Anisl 08059*	F: CCCTTCTCTCTGTGGAGTCG*	98 – 126	(CATC) 4	PET	13	“
	R: TGCTGCTATTCGAGCGTTTG					
*Anisl 00875*	F: TGACGCTCGAGTTGGTACAG*	142 – 172	(GCA) 8	NED	11	“
	R: GGTGGTGATGTTTACGCGAC					
*Anisl 07132*	F: ATCAGTGCCGAGTAGCATGG*	208 – 252	(ATTG) 7	FAM	12	“
	R: TTCAGGGTGCAAATGACGTG					
Multiplex 2						
*Anisl 00314*	F: CGTAGTGCTTCGCTTATCGC*	96 – 132	(GATA) 7	VIC	10	Mattiucci et al., 2019
	R: AGGGGATATGATCGAGATTAGACAG					
*Anisl 10535*	F: GTTTTGGGTTACCACCGACC*	125 – 146	(TTG) 9	PET	8	“
	R: GCAATGGGCAGTCATGGAAG					
*Anisl 00185*	F: CCGTGAACGCGATTCTCAAC*	185 – 209	(TTG) 7	FAM	9	“
	R: CCGCCTCCAAACAAACAAAC					
*Anisl 7*	F: TGATTGCGATTACTATTATTGTCAGT*	216 – 279	(TAT)8(TTA)5ctatttatt attatttaatattatcat(TTA)8tc(ATT)6	NED	18	Mladineo et al., 2017a
	R: GGTGGTGTCGTGACAGTGAT					
Multiplex 3						
*Anisl 4*	F: ACAAGGAATGCTCGTCGATATGA*	130 – 193	(TAA) 9	FAM	25	Mladineo et al., 2017b
	R: TCACTCTTGTCCAGCACTGC					
*Anisl 15*	F: GCAGACACTCATTCGCAGTC*	231 – 251	(CAAT) 7	NED	6	“
	R: GCTGATCCCTTGAAGTGGCT					
*Anisl 22*	F: TCAGAGGTGCGTAACGTTCA*	185 – 263	(TTA) 30	PET	24	“
	R: GCCACATCCATCCAAAAGAGC					

* = labelled primer. Fluorescent dyes: VIC = green; PET = red; NED = yellow, FAM = blue.

The same specimens were then genotyped for a total of 7 microsatellite loci (i.e. *Anisl 00314, Anisl 00875, Anisl 00185, Anisl 05784, Anisl 07132, Anisl 08059* and *Anisl 10535*), previously developed and validated in the three species of the *A. simplex* (s.l.) complex (Mattiucci et al., 2019; Bello et al., 2020). Additionally, the other 4 SSR loci (i.e. *Anisl 4, Anisl 7, Anisl 15* and *Anisl 22*) among those developed in Mladineo et al. (2017a) were here scored for the first time on specimens of the two parasite species. The amplification of microsatellite loci was carried out in three multiplex PCRs: *Anisl 05784, Anisl 08059, Anisl 00875* and *Anisl 07132* in Multiplex 1; *Anisl 00185, Anisl 00314, Anisl 10535* and *Anisl 7* in Multiplex 2; *Anisl 4, Anisl 15* and *Anisl 22* in Multiplex 3 (Table 2). The PCR conditions followed those described in Mattiucci et al. (2019).

To increase the number of diagnostic loci necessary to discriminate patterns of hybridization between the two species, additional nuclear gene loci, i.e. the superoxide dismutase (*Sod*) and the adenylate kinase (*Adk*) nDNA, were selected. Primers for these loci were designed for this work based on transcriptome sequences of *A. pegreffii* previously deposited by Palomba et al. (2022) using the online software program Primer3 (http://bioinfo.ut.ee/primer3-0.4.0/). PCR amplifications were performed using the primers SOD-F and SOD-R for the *Sod* nDNA gene and ADK-F and ADK-R for the *Adk* nDNA gene (see Table 2). Both PCRs were carried out in a 25 μL volume containing 1 μL of each 10 mM primer, 2 μL of 25 mM MgCl_2_ (Promega), 5 μL of 5 × buffer (Promega), 0.8 μL of 10 mM mix containing the four deoxynucleotide triphosphates (dNTPs, Promega), 5 U of Go-Taq Polymerase (Promega) and 2 μL of total DNA. The PCR conditions for the amplification of *Sod* nDNA were as follows: 95 °C for 2 min (initial denaturation), followed by 40 cycles at 95 °C for 30 s (denaturation), 57 °C for 45 s (annealing), 72 °C for 90 s (extension) and followed by post-amplification at 72 °C for 7 min. The PCR conditions for the amplification of *Adk* nDNA were as follows: 94 °C for 5 min, followed by 35 cycles of denaturation at 94 °C for 1 min, annealing at 60 °C for 1 min and extension at 72 °C for 1 min, with a final extension step at 72 °C for 1 min. The 2 new gene loci were thus validated on the same specimens (N = 431) of *A. pegreffii* and *A. simplex* (s.s.) that had previously been analysed using other nuclear markers. The purified PCR products from all markers were sequenced by an external company (Bio-Fab Research©).

Finally, all the specimens tested at those nuclear loci were sequenced at the mitochondrial *cox*2 gene (Mattiucci et al., 2014) using the primers 211 F and 210 R (Nadler and Hudspeth, 1998) (Table 2), following the procedures detailed in Mattiucci et al. (2014).

### Genetic data analysis

The sequences obtained from the N = 429 individuals at both mitochondrial and nuclear gene loci were edited and then aligned with reference sequences using the software ClustalX v.2. The same sequences were also analysed using Genemapper v.4.1 and PeakScanner v.2. (Applied Biosystems, Waltham, MA, USA). MICRO-CHECKER v.2.2.3 (Van Oosterhout et al., 2004) was used to check for genotyping errors. ARLEQUIN v.3.5 (Excoffier and Lischer, 2010) was used to calculate the genetic diversity indices observed at the 11 SSR loci, i.e. the observed heterozygosity (*Ho*), the expected heterozygosity (*He*) and the mean number of alleles per locus (A). This software also assessed the occurrence of Hardy−Weinberg Equilibrium (HWE) at each SSR locus through exact test outcomes, and it was also used to estimate the fixation index (*F_IS_*) from the genetic datasets acquired from the SSR loci in the analysed populations of the two *Anisakis* species. In addition, AMOVA and matrix of pairwise genetic differentiation (*Fst*) (Wright, 1949, 1965) were calculated using ARLEQUIN v.3.5 on microsatellite and nuclear single nucleotide polymorphism (SNP) data.

A model-based Bayesian clustering algorithm was implemented in STRUCTURE v.2.3.3 (Pritchard et al., 2000) using the genotypes at the diagnostic nuclear SNPs and diagnostic and partially diagnostic (between the 95% and 99%) SSR loci, in order to evaluate the admixture of pure lineages and to estimate mixing proportions between *A. simplex* (s.s.) and *A. pegreffii* in the analysed samples.

With the number of clusters (K) set between 1 and 3, 20 replicates of the analysis were carried out, each with 100 000 Markov chain Monte Carlo (MCMC) iterations and a burn-in period of 50 000 steps. The admixture model assumes that each individual has ancestry from one or more of K genetically distinct sources (Lawson et al., 2018). The best K value was identified using both the log probability of the data and the rate of change in the log probability of the data between successive K values as the optimality criteria (Evanno et al., 2005). A model-based Bayesian clustering method implemented in NEWHYBRIDS v.1.1 (Anderson and Thompson, 2002) was used to evaluate the most parsimonious allocation of samples. Specifically, the analysis tested for the presence of pure parental individuals (*A. simplex* (s.s.) or *A. pegreffii*), F1 hybrids and backcrossed individuals to both species. TCS haplotype network construction was carried out using PopART v.1.7 (Clement et al., 2002). The analysis was performed using the statistical parsimony procedure (95% parsimony connection limit), implemented in TCS v.1.21 (Templeton et al., 1992).

## Results

### *Genotyping* Anisakis *spp. specimens by* EF1 α-1 *and* Nas 10 *nDNA regions*

**Figure 2.**
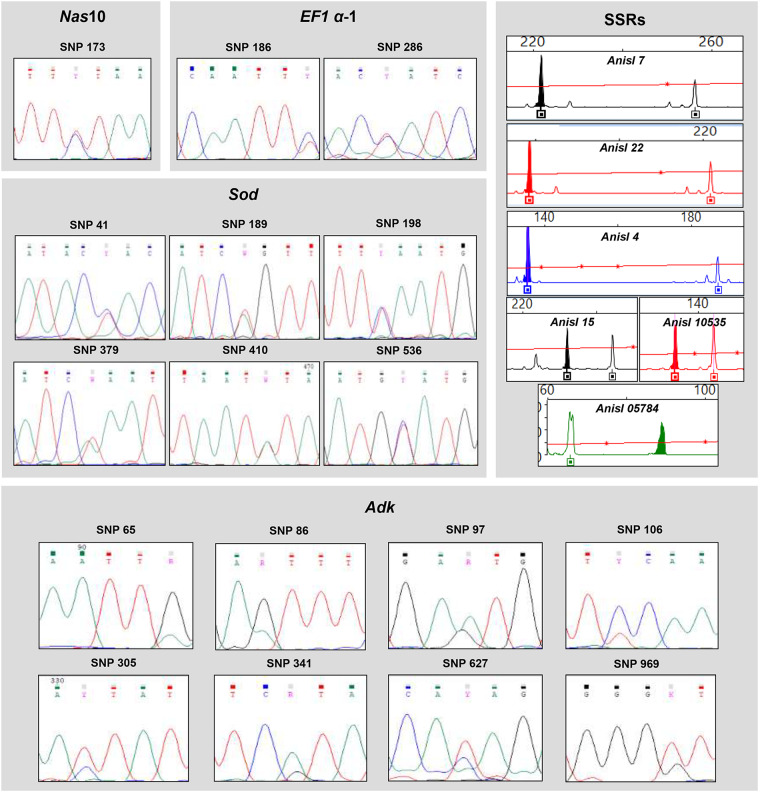
Chromatograms at the diagnostic SNPs in *Nas10, EF1 α-1, Sod* and *Adk* nDNA loci and electropherograms of the 5 diagnostic and partially diagnostic SSR loci (*Anisl 7, Anisl 22, Anisl 15, Anisl 4* and *Anisl 10535*), showing the heterozygous genotype having a double peak between *A. simplex* (s.s.) and *A. pegreffii* exhibited by admixed specimens between the two species.

Sequences of 409 bp from the nuclear *EF1 α-1* nDNA partial region were obtained for all 429 larval and adult specimens analysed in this study (Table 1). This fragment revealed 2 diagnostic SNPs distinguishing *A. pegreffii* from *A. simplex* (s.s.), as reported by Mattiucci et al. (2016). Based on this analysis, N = 340 specimens, homozygous for these SNPs, were assigned to *A. pegreffii*, whereas N = 70 specimens, homozygous for the alternative allele, were assigned to *A. simplex* (s.s.). Heterozygous genotypes at these diagnostic loci were found in N = 19 individuals (Fig. 2) from the sympatric areas here studied. A total of 4 sequences of the *EF1 α-1* nDNA region were deposited in GenBank under the accession numbers PV088038/39 for *A. pegreffii* and PV088040/41 for *A. simplex* (s.s.).

Similarly, a 451 bp sequence fragment of the *Nas10* nDNA region was obtained for all 429 specimens analysed. The sequences revealed 1 diagnostic SNP between *A. simplex* (s.s.) and *A. pegreffii* (see Palomba et al., 2020b). This position resulted homozygous CC in the *A. simplex* (s.s.) individuals (N = 70) from both its sympatric and allopatric populations, whereas it was always homozygous GG in *A. pegreffii* (N = 338) from its allopatric and sympatric populations here examined. However, patterns of heterozygote genotypes at that diagnostic position were found in those individuals (N = 19) also resulted heterozygous at the *EF1 α-1* nDNA as above described, plus 2 specimens; all of them collected from the sympatric areas here studied (Fig. 2).

A total of 4 sequences of the *Nas10* nDNA region were deposited in GenBank under the following accession numbers PV088042/43 for *A. pegreffii* and PV088044/45 for *A. simplex* (s.s.).

### *Diagnostic SNPs at the novel* Sod *and* Adk *nDNA gene loci*

**Figure 3.**
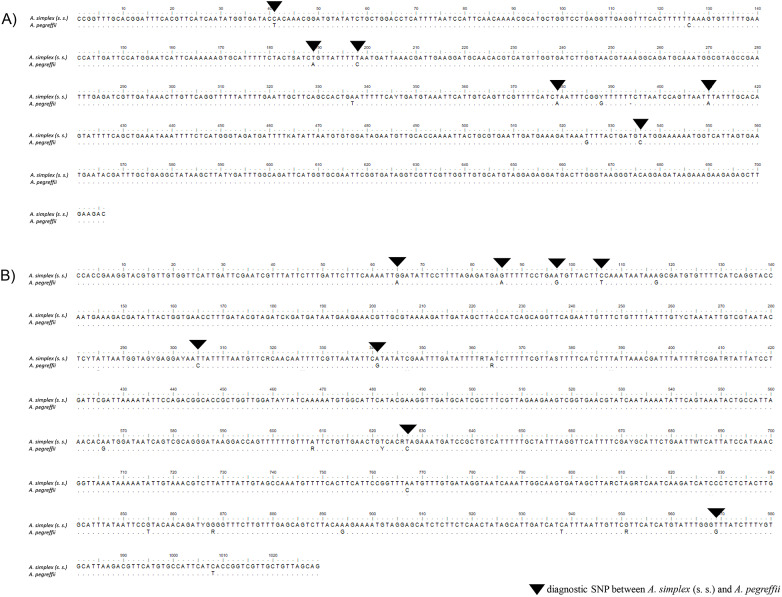
(A) Alignment of the region of the *Sod* nDNA, showing the 100% diagnostic positions: 6 SNPs (black triangles) between *A. simplex* (s.s.) and *A. pegreffii.* (B) Alignment of the region of the *Adk* nDNA, showing the 100% diagnostic positions: 8 SNPs (black triangles) between *A. simplex* (s.s.) and *A. pegreffii.*

Fixed diagnostic nucleotide positions capable of discriminating between the two species were detected at both *Sod* and *Adk* nDNA gene loci (Fig. 3A and Fig. 3B). In particular, a 706 bp fragment of the *Sod* nDNA region was obtained for all of the 429 specimens analysed. This fragment revealed the presence of 6 fixed alternative (i.e. diagnostic) SNPs between *A. pegreffii* and *A. simplex* (s.s.) (Fig. 3A). Based on these positions, N = 340 specimens, being homozygous for the 6 diagnostic SNPs, were assigned to *A. pegreffii*, whereas N = 78 specimens, also being homozygous for the alternative SNPs, were assigned to *A. simplex* (s.s.). Finally, a total of N = 11 specimens showed a heterozygous genotype at all 6 diagnostic positions (shown in Fig. 2). These individuals were among the N = 21 also observed as heterozygous at the diagnostic SNPs found at the *Nas10* nDNA. A total of 4 sequences of the *Sod* nDNA region were deposited in GenBank under the accession numbers PV088050/51 for *A. pegreffii* and PV088052/53 for *A. simplex* (s.s.).

Similarly, a 1062 bp fragment of the *Adk* nDNA region was obtained from the same N = 429 allopatric and sympatric specimens of the two targeted species. This fragment revealed the presence of 8 diagnostic SNPs between *A. pegreffii* and *A. simplex* (s.s.), showing fixed alternative nucleotides between the two species (Fig. 3B). Based on these diagnostic positions, N = 338 specimens were assigned to *A. pegreffii*, whereas N = 71 specimens, showing the opposite SNPs, were assigned to *A. simplex* (s.s.). Among the total number of tested specimens, N = 20 individuals showed a heterozygous genotype at all the 8 diagnostic SNPs (shown in Fig. 2) among the N = 21 heterozygous found at the diagnostic SNPs found at the *Nas10* nDNA. A total of 4 sequences of the *Adk* nDNA region were deposited in GenBank under the accession numbers PV088046/47 for *A. pegreffii* and PV088048/49 for *A. simplex* (s.s.).

### Novel diagnostic SSR DNA loci

Finally, the same 429 individual specimens were also genotyped at 11 scored SSR DNA loci within the metapopulations of *A. pegreffii* and *A. simplex* (s.s.) (Table 3). Differences in the overall genetic diversity values of the different populations of the two species were observed, based on both the A value and the *He* value per locus (Table 3). All the microsatellite loci scored in the present study were polymorphic, with the total number of alleles varying in *A. pegreffii* between 5 (e.g. at loci *Anisl* 7 and *Anisl 22*) and up to 13 at the newly scored locus *Anisl 4* (Table 3). In *A. pegreffii*, the A value across the 11 SSR loci was 8.7. In *A. simplex* (s.s.), the A value was 9.6 with the newly scored locus *Anisl 2*2 showing the highest number of alleles N = 25, while the minimum number of alleles was found at the loci *Anisl 10535* and *Anisl 8059* (Table 3).

**Table 3.** Genetic diversity, at 11 SSR loci, in populations of *A. pegreffii* and *A. simplex* (s.s.) analysed in the present study. No estimates were possible for the locus *Anisl 15* because it was monomorphic in *A. pegreffii* populations

		*A. pegreffii*	*A. simplex* (s.s.)
Locus			
*Anisl 00185*	N	157	61
	*Ho*	0.69	0.68
	*He*	0.78	0.72
	P value	0.03	0.16
	A	8	7
*Anisl 00314*	N	158	62
	*Ho*	0.39	0.19
	*He*	0.77	0.79
	P value	***	***
	A	7	7
*Anisl 10535*	N	158	62
	*Ho*	0.34	0.12
	*He*	0.33	0.16
	P value	0.79	0.03
	A	7	5
*Anisl 07132*	N	158	63
	*Ho*	0.71	0.53
	*He*	0.75	0.73
	P value	0.39	0.02
	A	10	9
*Anisl 05784*	N	158	61
	*Ho*	0.81	0.57
	*He*	0.78	0.73
	P value	0.08	0.02
	A	11	10
*Anisl 08059*	N	157	61
	*Ho*	0.78	0.37
	*He*	0.85	0.41
	P value	0.04	0.02
	A	13	5
*Anisl 00875*	N	158	61
	*Ho*	0.43	0.55
	*He*	0.44	0.70
	P value	0.18	0.06
	A	8	9
*Anisl 7*	N	155	50
	*Ho*	0.20	0.46
	*He*	0.63	0.67
	P value	***	***
	A	5	8
*Anisl 4*	N	154	60
	*Ho*	0.39	0.56
	*He*	0.73	0.91
	P value	***	***
	A	13	15
*Anisl 22*	N	155	63
	*Ho*	0.32	0.47
	*He*	0.61	0.93
	P value	***	***
	A	5	25
*Anisl 15*	N	–	62
	*Ho*	–	0.40
	*He*	–	0.70
	P value	–	***
	A	–	6

N, number of genotyped specimens at each locus; *Ho*, observed heterozygosity; *He*, expected heterozygosity; A, number of alleles detected at each locus; P value, significance (*P*<0.05) of the deviation from HWE expectation.

*** *P*≪0.01.

No significant deviations between *Ho* and *He* were observed at loci *Anisl 10185* and *Anisl 08059* in all analysed samples of *A. pegreffii*. Similarly, in *A. simplex* (s.s.), no significant departures from HWE were observed at loci *Anisl 10535, Anisl 07132, Anisl 05784* and *Ansil 08059* (Table 3). Significant deviations from HWE were found in both species at loci *Anisl 00185* and *Anisl 7*, as well as at the newly tested loci *Anisl 4, Anisl 22* and *Anisl 15* (Table 3). These loci showed a marked excess of homozygotes in the two species (Table 3), with positive *F_IS_* values (Suppl. Figure 1a). Generally, positive *F_IS_* values indicate an excess of homozygote genotypes at the selected loci, while negative values indicate an excess of heterozygotes from the expected HWE (Suppl. Figure 1a). When dividing the genotypes at these 5 loci into adult male and female worms, they were found to be in HWE in the female genotypes (Table 4). The male individuals of both species were homozygous at those loci (*F_IS_* = 1) (Suppl. Figure 1b), likely due to their hemizygosity state, indicative of X-linked inheritance. At the newly scored locus *Anisl 15*, the specimens of *A. pegreffii* were monomorphic (Table 4), showing only the allele 231 (Suppl. Table 1), preventing the demonstration of sex-linkage in *A. pegreffii*. In contrast, this locus was sex-linked in *A. simplex* (s.s.), as were the other 2 newly tested SSR loci, i.e. *Anisl 4* and *Anisl 22* in both species. Accordingly, no significant departures from the HWE were found between the expected homozygous and heterozygous females at these loci, nor at locus *Anisl 7* in the two *Anisakis* spp. (Table 4). Analogously, no significant departure from HWE was found at the locus in any of the populations of *A. pegreffii* (Table 4).

**Table 4.** Genetic diversity at the 5 sex-linked loci (i.e. *Anisl 00314, Anisl 7, Anisl 4, Anisl 22* and *Anisl 15*) in *A. pegreffii* and *A. simplex* (s.s.) estimated in adult female specimens. Estimates at the locus *Anisl 15* were not possible because it is monomorphic in *A. pegreffii*

Locus		*A. pegreffii*	*A. simplex* (s.s.)
		♀♀	♀♀
*Anisl 00314*	N	28	16
	*Ho*	0.75	0.43
	*He*	0.79	0.75
	P value	0.67	0.03
*Anisl 7*	N	28	16
	*Ho*	0.42	0.75
	*He*	0.53	0.72
	P value	0.37	0.39
*Anisl 4*	N	28	17
	*Ho*	0.75	0.88
	*He*	0.70	0.92
	P value	0.99	0.52
*Anisl 22*	N	28	17
	*Ho*	0.60	0.88
	*He*	0.52	0.93
	P value	0.34	0.26
*Anisl 15*	N	–	17
	*Ho*	–	0.52
	*He*	–	0.74
	P value	–	0.02

N, number of genotyped specimens at each locus; *Ho*, observed heterozygosity; *He*, expected heterozygosity; P value, significance (*P*<0.05) of the deviation from HWE expectation.

**Figure 4.**
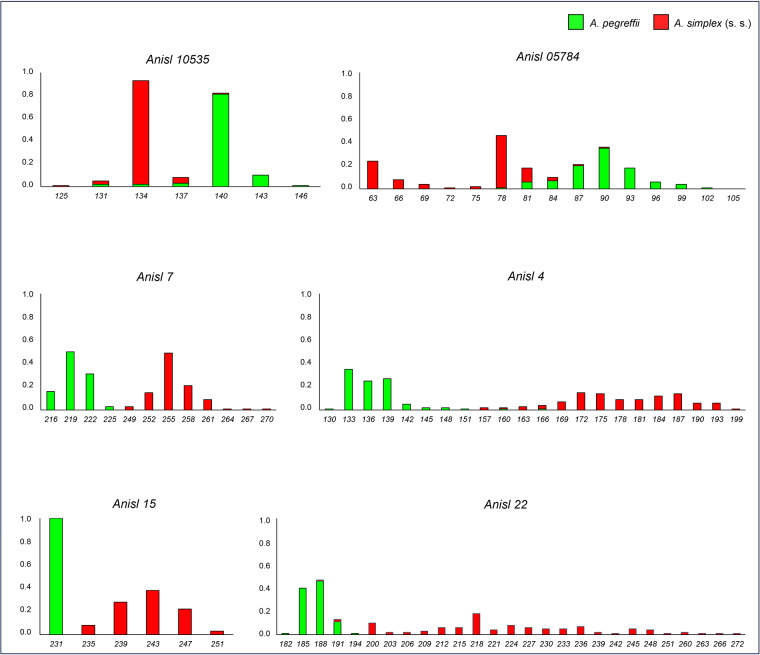
Distribution of allele frequencies at the partially diagnostic and 100% diagnostic SSR loci between *A. pegreffii* (green) and *A. simplex* (s.s.) (red).

Allele frequencies estimated in *A. pegreffii* and *A. simplex* (s.s.) are reported in Suppl. Table 1. For the sex-linked loci (i.e. *Anisl 00314, Anisl 7, Anisl 4, Anisl 15* and *Anisl 22*), allele frequencies were calculated considering the hemizygosity of male specimens, with males considered monoallelic and females biallelic, according to Mattiucci et al. (2019). Among the newly tested SSR loci, *Anisl 4, Anisl 15* and *Anisl 22* showed significant differences in allele frequencies between the two sibling species (Fig. 4). Indeed, *Anisl 15* and *Anisl 7* were 100% diagnostic between the two species. A total of N = 11 specimens showed at these SSR loci both the allele of the parental taxon *A. simplex* (s.s.) and that of *A. pegreffii* (Fig. 2).

### Identification of genetic admixture between the two species by Bayesian multi-marker genotyping approach

**Figure 5.**
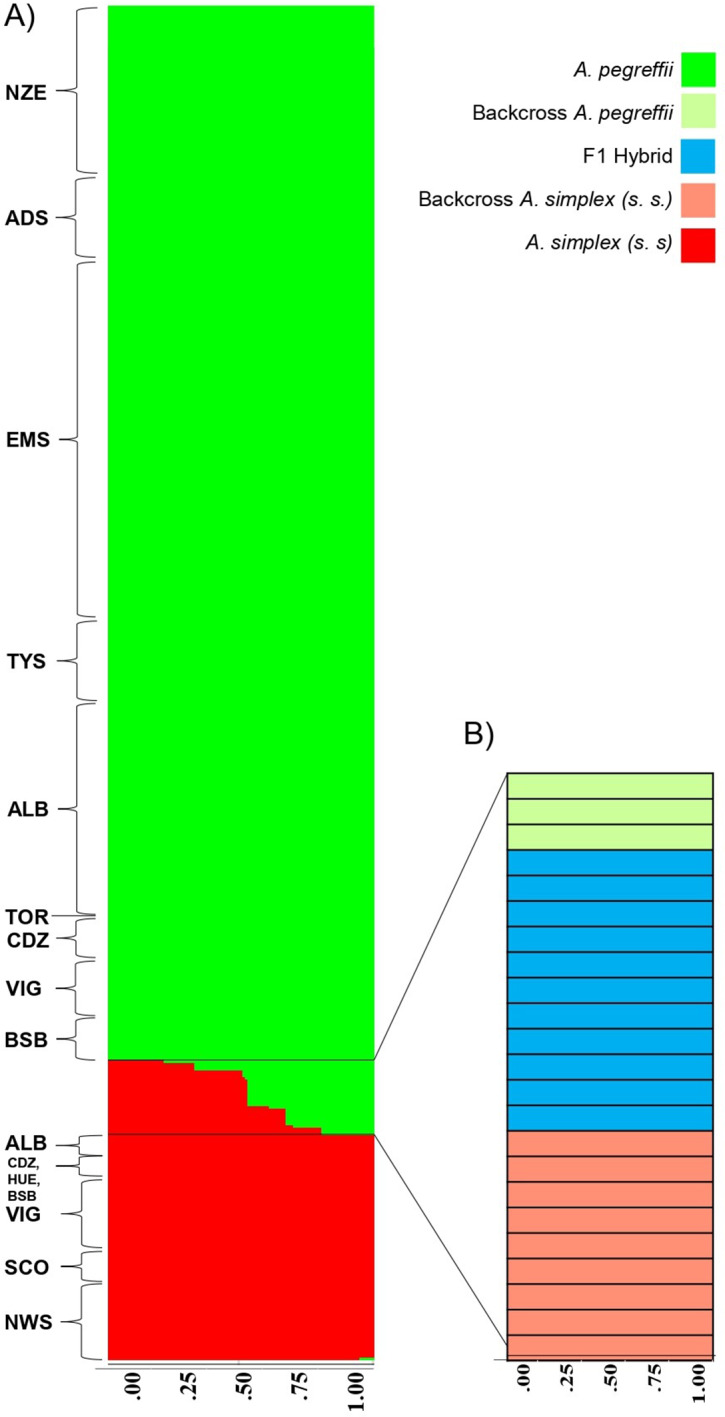
(A) Percentage contribution (Q) of each specimen of *A. pegreffii* and *A. simplex* (s.s.) to each cluster following a Bayesian clustering analysis implemented in STRUCTURE on 17 diagnostic SNPs at nDNA gene loci (*EF1 α-1, Nas10, Sod* and *Adk*) and at the SSR loci (i.e. *Anisl 10535* and *Anisl 05784*; sex-linked loci were excluded to include male specimens in the elaboration), with K = 2. (B) The posterior probability of assignment to 1 of 5 genotype classes following a Bayesian clustering analysis implemented in NEWHYBRIDS. Those specimens with evidence of admixture were assigned to pure *A. pegreffii* and *A. simplex* (s.s.), F1 hybrids or backcrossed categories with a 100% posterior probability value of assignment.

The results inferred from the Bayesian clustering algorithm of all 429 genotyped specimens performed by STRUCTURE 2.3.3., using the N = 17 100% diagnostic SNPs found in the nuclear loci, *plus* the genotypes found at the partially diagnostic loci (between 95% and 99%). SSR loci between the two parental species, i.e. *Anisl 10535* and *Anisl 05784*, of the 429 specimens are presented in Fig. 5A. We excluded sex-linked loci (i.e. *Anisl 15* and *Anisl 7*), despite being 100% diagnostic between the two parental taxa, in order to include in the analysis also the male specimens. Using the K = 2 clustering option, implemented by the Evanno method, the dataset best fit a ΔK = 48.765.

According to the STRUCTURE analysis, it was possible to assign with a posterior probability of assignment of 100%, N = 406 specimens to the two pure parental taxa: *A. pegreffii* or *A. simplex* (s.s.). In particular, N = 337 individuals were identified as *A. pegreffii*, including both adults and L3 of *Anisakis* (Fig. 5A; Table 5). Similarly, N = 69 individuals were assigned to *A. simplex* (s.s.) with a posterior probability of *p* = 100% (Fig. 5A; Table 5). Finally, a total of N = 23 individuals, as evidenced by the results obtained by the single nuclear marker above reported, showed admixtures in their nuclear genome, including L3 and adult specimens collected from the Atlantic Iberian and Alboran Sea coast (Fig. 5A; Table 5). In the case of these admixed individuals, NEWHYBRIDS, carried out on the same genotypes observed at all the nuclear gene loci tested, disclosed that N = 11 individuals were F1 hybrids between the two parental taxa, with 100% posterior probability (Fig. 5B). Additionally, NEWHYBRIDS showed that among the remaining 12 individuals showing admixture genome in the STRUCTURE analysis, N = 3 individuals were backcrosses to *A. pegreffii*, while N = 9 were backcrossed to *A. simplex* (s.s.) (Fig. 5B; Table 5). Only 1 adult female resulted to be F1 hybrid genotype, while all the backcrossed individuals were L3-stage larvae collected from fish hosts (Table 5). Concordant results between STRUCTURE and NEWHYBRIDS were achieved in the assignment of the remaining 406 specimens tested to the two pure parental genomes, *A. pegreffii* or *A. simplex* (s.s.), with 100% posterior probability (Table 5).

**Table 5.** Number of specimens assigned to different classes of introgression or to parental species: Pure parental *A. pegreffii*; Pure parental *A. simplex* (s.s.); F1 Hybrid; Backcross × *A. pegreffii*; Backcross × *A. simplex* (s.s.), on the basis of a Bayesian clustering approach implemented in STRUCTURE and NEWHYBRIDS combining the diagnostic SNPs at nDNA gene loci and SSR loci. The number of specimens by mtDNA *cox*2 gene locus is also represented

Area	Host species	Multinuclear genotyping assignment					mtDNA *cox*2	
		Pure parental *A. pegreffii*	Pure parental *A. simplex* (s.s.)	F1 Hybrid	Backcross *A. pegreffii*	Backcross *A. simplex* (s.s.)	*A. pegreffii*	*A. simplex* (s.s.)
ADS	*Lophius piscatorius*	2	–	–	–	–	2	–
	*Merluccius merluccius*	2	–	–	–	–	–	2
	*Scomber scombrus*	3	–	–	–	–	3	–
	*Trachurus trachurus*	1	–	–	–	–	1	–
	*Stenella coeruleoalba*	18	–	–	–	–	18	–
TYS	*Lepidopus caudatus*	21	–	–	–	–	21	–
	*Merluccius merluccius*	2	–	–	–	–	–	2
	*Scomber scombrus*	4	–	–	–	–	4	–
EMS	*Merluccius merluccius*	102	–	–	–	–	96	6
	*Phycis blennoides*	20	–	–	–	–	14	6
TOR	*Merluccius merluccius*	1	1	–	–	–	1	1
ALB	*Merluccius merluccius*	60	5	1	1	1	61	7
	*Trachurus trachurus*	3	–	–	–	–	3	–
CDZ	*Merluccius merluccius*	12	2	–	1	1	13	3
HUE	*Merluccius merluccius*	15	1	2	–	–	15	3
VIG	*Merluccius merluccius*	–	1	1	1	3	1	5
	*Delphinus delphis*	19	11	–	–	–	19	11
	*Globicephala melas*	–	3	–	–	–	–	3
	*Phocoena phocoena*	–	7	1	–	1	2	7
BSB	*Micromesistius poutassou*	2	3	5	–	3	4	9
	*Trachurus trachurus*	–	–	1	–	–	–	1
SCO	*Lagenorhynchus albirostris*	–	5	–	–	–	–	5
	*Stenella coeruleoalba*	–	5	–	–	–	–	5
NWS	*Clupea harengus*	–	13	–	–	–	–	13
	*Micromesistius poutassou*	–	7	–	–	–	–	7
	*Scomber scombrus*	–	5	–	–	–	–	5
NZE	*Globicephala melas*	50	–	–	–	–	50	–
	Tot:	337	69	11	3	9	328	101

### Mitochondrial inference of the pure and hybrid specimens

**Figure 6.**
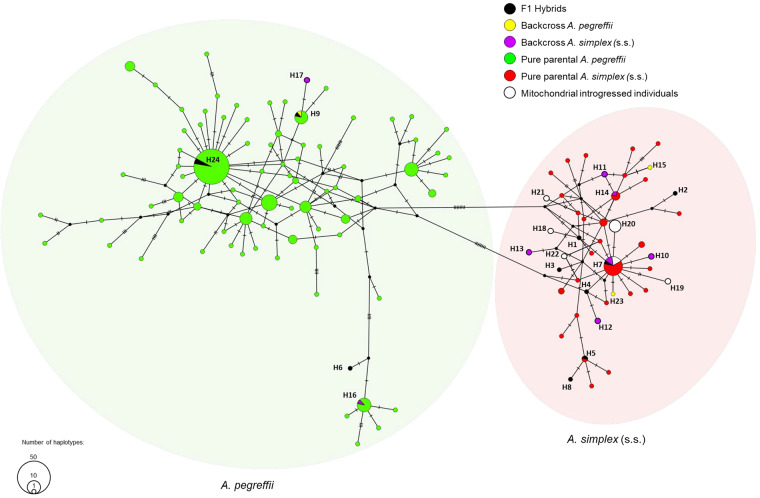
Statistical parsimony haplotype network (TCS) among mtDNA *cox*2 haplotypes showed by pure specimens of the two parental species *A. pegreffii* and *A. simplex* (s.s.) and by their hybrid individuals, *plus* the specimens from ATYS, ADS and EMS sea waters showing mitochondrial introgression. Circles suggest the two different haplogroups represented by the parental species (*A. pegreffii*: light green, *A. simplex* (s.s.): light red).

The results obtained from the mitochondrial DNA (mtDNA) *cox*2 sequence analysis of all those N = 429 specimens studied at the nuclear gene loci are reported in Table 5. The haplotype parsimony network (TCS) inferred from mtDNA *cox*2 sequences of all the specimens, as recognized by the STRUCTURE and NEWHYBRIDS analyses, showed the presence of two major haplogroups represented by the two pure parental taxa (Fig. 6). The TCS analysis of mtDNA *cox*2 haplotypes resulted in a star-like tree for both haplogroups (Fig. 6). The haplotypes H23, H26, H30 and H31 were found as the most frequent in the pure parental specimens of *A. pegreffii* recognized by the STRUCTURE analysis of the nuclear genome. Analogously, specimens showing the nuclear genome of *A. simplex* (s.s.) recognized by STRUCTURE inference mostly exhibited the haplotypes H7 and H14 shared by all metapopulations of the parasite species (Fig. 6). Full concordance with nuclear genotyping was obtained in the recognition of all pure specimens collected from the allopatric areas, i.e. of *A. pegreffii* from off New Zealand coast and of *A. simplex* (s.s.) collected from the Norwegian Sea.

The TCS network also revealed that among the confirmed F1 hybrids detected by NEWHYBRIDS, N = 4 had *A. pegreffii* maternal inheritance (Fig. 6; Table 5), whereas N = 7 had *A. simplex* (s.s.) maternal inheritance (Fig. 6; Table 5). The haplotypes shown by these F1 individuals were also observed in the respective parental taxa: H1, H2, H3, H4, H5, H7 and H8 in *A. simplex* (s.s) and H6, H9 and H21 in *A. pegreffii*. Additionally, 1 backcross of *A. pegreffii* had *A. pegreffii* maternal inheritance, showing the haplotype H9. Other larval backcrosses to *A. pegreffii* found in *Merluccius merluccius* from the Alboran Sea and Cadiz had *A. simplex* (s.s.) maternal inheritance showing haplotypes H15 and H22, which were shared with other pure specimens of *A. simplex* (s.s.). Most backcrosses of *A. simplex* (s.s.), observed as L3 in fish from NE Atlantic waters, had *A. simplex* (s.s.) maternal inheritance, showing haplotypes H7, H10, H11, H12, H13 and H14, while 2 backcrosses of *A. simplex* (s.s.) had *A. pegreffii* maternal inheritance showing haplotypes H16 and H17.

Interestingly, among the N = 337 individuals assigned to *A. pegreffii* with 100% posterior probability value by STRUCTURE and NEWHYBRIDS, N = 16 specimens collected from the Adriatic, Ionian and Tyrrhenian Sea showed a mito-nuclear discordance. Indeed, the mtDNA *cox*2 of these specimens belonged to the *A. simplex* (s.s.) haplogroup (Fig. 6). In particular, 4 individuals shared the same H7 haplotype previously observed in other *A. simplex* (s.s.) specimens from Atlantic Iberian coast (Murphy et al., 2010; Klapper et al., 2016; Pons-Bordas et al., 2020; Simsek et al., 2020; Moratal et al., 2023) and Adriatic Sea waters (Buselic et al., 2015). The remaining individuals (N = 12) exhibited the haplotypes H18, H19, H20, H21 and H22 (Fig. 6). Among these haplotypes, H20 was previously observed in the Mediterranean Sea waters (Buselic et al., 2015), and their sequences matched (100%) with those obtained from the Adriatic Sea and Tunisian coast, whereas H18, H19, H21 and H22 resulted as new haplotypes, not matching with other sequences deposited in GenBank (Fig. 6). A total of 4 sequences of the *cox2* mtDNA were deposited in GenBank under the accession numbers PV088034/35 for *A. pegreffii* and PV088036/37for *A. simplex* (s.s.).

### Interspecific and intraspecific genetic differentiation inferred from nuclear and mitochondrial data

Excluding the sex-linked loci due to male hemizygosity, the AMOVA analysis of all nuclear gene loci analysed in this study revealed that, for both species, most of the genetic variation was significantly allocated within individuals (≈72% and ≈95%). Notable variation was found among individuals within populations, with *FIT* = 0.27 (P=0.0000) in *A. pegreffii* and *FIT* = 0.05 (P=0.3206) in *A. simplex* (s.s.) (data not shown).

A high level of genetic differentiation was found at the interspecific level between the metapopulations of *A. pegreffii* and *A. simplex* (s.s.), based on the SSR loci and the SNPs at the nDNA scored, with an average *Fst* ≈ 0.95. A significant genetic differentiation was also observed between backcrosses to *A. simplex* (s.s.) vs pure parental *A. pegreffii* (on average, *Fst* ≈ 0.64), as well as between backcrosses to *A. pegreffii* vs pure parental *A. simplex* (s.s.) (on average, *Fst* ≈ 0.60) (data not shown). Lower but significant differentiation values were observed between F1 hybrids and the parental taxa (*Fst* ≈ 0.45 and *Fst* ≈ 0.49, respectively, vs *A. pegreffii* and *A. simplex* (s.s.)).

Similarly, at the cytoplasmic level, a significantly high level of differentiation (*Fst* *=* 0.73; P<0.0000) was inferred from the mtDNA *cox*2 dataset, between the two haplogroups (i.e. *A. pegreffii* and *A. simplex* s.s.).

## Discussion

### *Novel nuclear diagnostic markers between* A. pegreffii *and* A. simplex *(s.s.)*

Efforts have been recently made to pinpoint genome regions and individual SNPs that are diagnostic (i.e. 100% alternative) between the three sibling species in the *A. simplex* (s.l.) complex (i.e. *A. simplex* (s.s.), *A. pegreffii* and *A. berlandi*) to study their geographical contact zones (Mattiucci et al., 2016, 2019; Gómez-Mateos et al., 2020; Bello et al., 2021). For the first time, we identified and validated genetic diagnostic variants in novel autosomal nDNA of *A. simplex* (s.s.) and *A. pegreffii*, using a large number of specimens from both allopatric populations and the contact zones along the Atlantic Iberian coast and Mediterranean Sea waters. Indeed, 14 fixed diagnostic alternative SNPs were discovered in 2 newly analysed autosomal gene loci, i.e. *Sod* and *Adk* nDNA (Fig. 3A and Fig. 3B). In addition, further SSR loci, including autosomal and sex-linked ones, among those developed and studied in *A. simplex* (s.l.) (Mattiucci et al., 2019; Bello et al., 2020), were found to be of diagnostic value for distinguishing the two parental taxa here studied. In particular, the loci *Anisl* 4, *Anisl* 7, *Anisl 22* and *Anisl 15* were found to have 99% or 100% of diagnostic value between the two taxa (Fig. 4). Similarly, among these loci, *Anisl 15* showed also fixed alternative alleles between *A. berlandi* and *A. pegreffii* (Bello et al., 2021). Finally, *Anisl 10535* and *Anisl 05784* have been confirmed as partially diagnostic between the two taxa, as previously observed (Mattiucci et al., 2019).

### Novel sex-linked SSR loci

The validation of the newly scored SSR loci *Anisl 4, Anisl 22*, and *Anisl 15* in a large sample of *A. simplex* (s.s.) individuals (N = 69) revealed the existence of additional sex-linked microsatellite loci in these species (i.e. *Anisl 00314* and *Anisl 7*) (Mattiucci et al., 2019). Indeed, while loci *Anisl 4* and *Anisl 22* were previously found to be sex-linked in *A. pegreffii* (Bello et al., 2021), the same loci, along the *Anisl 15*, were identified for the first time as sex-linked in *A. simplex* (s.s.) in this study.

Sex-linked loci are genes located on the X chromosome. In our sample, which included both male and female adult specimens, we verified the potential occurrence of sex-linkage in the newly scored SSR loci. They inherit the chromosome(s) from the mother only, lacking the paternal chromosomal counterpart, thus possessing just one copy of that gene. So far, the proportion of sex-linked microsatellites in the whole genome of the *A. pegreffii* and *A. simplex* (s.s.) can be estimated at about 33%, as found in our previous SSR analysis in *A. berlandi* (Bello et al., 2021). XX/XO female/male sex determination appears to be common among nematodes (Harvey and Viney, 2001), since it is frequently reported that males have 1 less chromosome than females (Walton, 1940). To date, preliminary cytogenetic studies recently carried out on *A. pegreffii* (Moratal et al., 2023) have revealed that *A. pegreffii* seems to possess a diploid number of 2n = 18, likely consisting of autosomal holocentric chromosomes (Moratal et al., 2023). However, no specific data on sexual chromosomes were provided by the same Authors. Additionally, by using a telomeric probe (TTAGGC)n in *A. pegreffii*, Moratal et al. (2023) identified the presence of a conserved telomeric sequence, with variability in signal intensity at the telomeric ends of chromosomes. Hypervariability in the length of telomeric repeats is not unusual in animals (Rocco et al., 2001), likely due to the low copy number of DNA repeats and the non-clustered organization of the tandem repeats (Moratal et al., 2023). It has been also hypothesized that the low number of chromosomes found in some cells of *A. pegreffii* is due to the loss of chromosomes during the metaphase (Moratal et al., 2023). However, no data concerning this phenomenon were provided by the same Authors. Further cytological work, combined with short and long-read sequencing technology, would shed light on whole chromosome-assemblage genomes, including sex chromosomes of the anisakid parasites, and their evolutionary significance.

In addition, the possible use of sex-linked loci for female/male identification has been recently discussed (Bello et al., 2021), and the finding of the new sex-linked loci, here achieved, will offer in future studies a higher-resolution power for their use in distinguishing male and female worms of these parasites, at any life-history stage.

### Distribution of different patterns of nuclear and mitochondrial introgression

According to the biological species concept (Mayr, 1963; Arnold, 1997), which defines species as reproductively isolated, interspecific hybrids should be extremely rare. In reality, the evolutionary boundaries between species are permeable, with some estimates suggesting that hybridization may occur in up to 10% of animal species and 25% in plants (Schwenk et al., 2008). The ubiquity of natural hybridization in terrestrial environments suggests it as a major driving force in evolutionary biology (Abbott et al., 2013), even if it can also lead to evolutionary dead ends and genetic swamping if hybrids are rare and isolated (Porretta and Canestrelli, 2023). Previously, naturally occurring hybrids among parasites were thought to be quite rare, but next-generation sequencing (NGS) analyses have recently enabled the detection of different patterns of natural hybridization in closely related parasite species (Criscione et al., 2007; Steinauer et al., 2008; Huyse et al., 2009; Chaudhry et al., 2015; Hecht et al., 2016; Leger and Webster, 2017; Calvani and Slapeta, 2020), as well as in parasites’ vectors (Weetman et al., 2014; Mancini et al., 2015; Caputo et al., 2021). Most of these studies have been carried out in contact zones, where the biogeographic distribution of these parasite or vector species overlaps. The two *Anisakis* species here studied in their contact zone are genetically well differentiated, as evidenced by the high *Fst* values observed at both nuclear and mitochondrial levels. This implies that multiple markers are required to characterize their hybrids’ gene pools. With multiple diagnostic nuclear markers, the probability of misclassification of an individual into an alternative category is very low and can be estimated by Mendelian principles. Indeed, when a backcrossing event is hypothesized to occur with the same parental species, several diagnostic nuclear markers may allow the identification of hybrid origin up to the third generation of backcrossing (fourth-generational hybrids). Thus, the multilocus assay used in the present study, employing a wider set of diagnostic SNPs (N = 17), was able to capture patterns of admixture reflection between the parental genomes. In fact, the NEWHYBRIDS analysis clearly shows that interspecific hybridization between the two species has led to genetic introgression of parental lineages (Fig. 5B). The presence of first- and later-generation hybrids among specimens of *A. pegreffii* and *A. simplex* (s.s.) from the contact zone of the Atlantic Iberian coast and the Alboran Sea indicates that recent introgression has occurred, demonstrating contemporary interspecific gene exchange. The combined analysis of microsatellites and SNP at nuclear loci indicate symmetric patterns of genomic hybridization, with approximately 5% of admixture between the two interacting species. It would be also interesting to test by a multigenotyping approach, whether gene introgression between *A. pegreffii* and *A. simplex* (s.s.) exhibits similar rates in other sympatric areas of their distribution (Mattiucci et al., 2018; Ding et al., 2022), potentially reflecting the species’ evolutionary history.

To date, introgression has not been reported between the two more phylogenetically divergent taxa of the *A. simplex* (s.l.) complex, *A. pegreffii* and *A. berlandi*. Indeed, only F1 hybrids, but not later generations of hybrid individuals, have been found between them in their overlapping geographical range in New Zealand waters (Bello et al., 2021).

### Ecological and evolutionary implications of the hybridization and mitochondrial introgression

In parasites, hybridization and introgression could have microevolutionary and ecological implications, including potential emerging public health concerns (Webster et al., 2013; Huyse et al., 2013; Boissier et al., 2015; Chaudhry et al., 2015). In our samples, the presence of hybrids appears to be driven by both ecological and evolutionary factors.

The achieved results suggest that there is a strong tendency for hybridization to take place between the two parental species when they greatly differ in abundance, and a tendency of the F1 hybrids to cross with the parental females at the respective limits of the geographical distribution of the two species. Although conclusions are only tentative, because the number of hybrid categories discovered is small with respect to the parental specimens, the data suggest a density-dependent mating pattern. The scarcity of the rarer species along the latitude distribution of the two species may be an important factor in increasing the likelihood of interspecific hybridization. Indeed, the numerical disparity between adult specimens of *A. pegreffii* and *A. simplex* (s.s.) has been generally observed in the contact zone here studied (Levsen et al., 2018; Cipriani et al., 2022) along a latitudinal gradient, co-infecting the same individual definitive hosts. The ecological conditions required by the two species shift in this geographic area, initially favouring *A. pegreffii* and, moving northward, *A. simplex* (s.s.). Stochastic processes related to demographic disparities between the two interacting species at the limit of their respective geographic distribution in NE Atlantic waters – specifically, the Bay of Biscay for *A. pegreffii* and conversely, the Morocco Atlantic coast for the species *A. simplex* (s.s.), where this species has been rarely detected at its larval stage (Mattiucci et al., 2018) – could occur.

**Figure 7.**
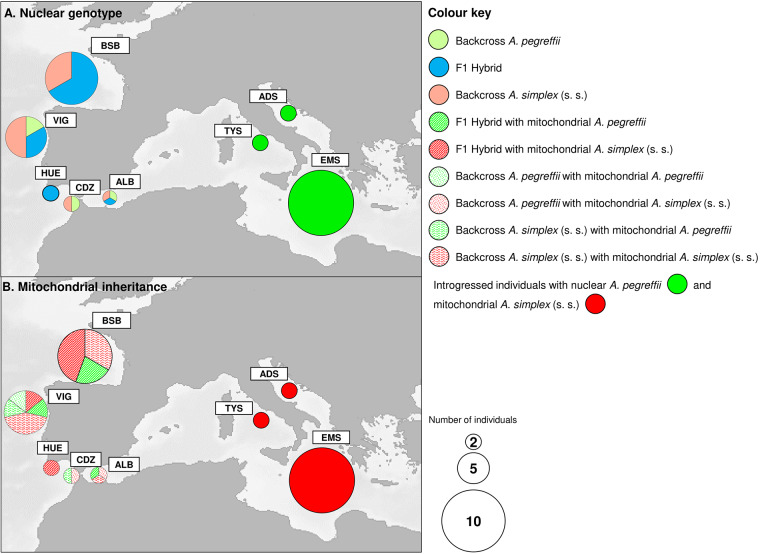
Spatial distribution and relative proportions of the F1 hybrids, backcrosses and mitochondrial introgressed specimens detected, in the present study, by multi-markers approach according to their nuclear genotype (A) as inferred from STRUCTURE and NEWHYBRIDS (see FIGURE 5), and mithocondrial genes (B).

The numerosity of the two species in their hosts follows this pattern. Under these circumstances, the syntopic disparity of the two species occurring at the adult stage in a definitive host from a contact area may promote heterospecific mating pairings. Indeed, among the F1 and backcross individuals found in the northern part of the sympatric area here studied, most of them have shown a mitochondrial genome of *A. pegreffii*, indicating that they were originated from females at the geographic limit of the parasite species distribution (Diez et al., 2022; Díez et al., 2024) (Fig. 7B). The opposite trend was observed in the southern part of the sympatric area (i.e. Portuguese and Alboran Sea coast), from where most of the F1 and backcross individuals have shown *A. simplex* (s.s.) mitochondrial genome, indicating females of the species at its limit of distribution (Fig. 7B).

In addition, the combined effect of different proportions of mature worms of the two species coinfecting the same definitive host in a contact area, coupled with their differential timing of sexual maturity, could be at the base of occasional hybridization events. Indeed, a possible differential reproductive timing could influence the fertilization success in the two closely related species, by preventing interspecific mating as a *pre-copula* mechanism of reproductive isolation. For instance, *in vitro* culture experimental works, carried out from L3 to reproductive adult stage of the two species, have demonstrated that females of *A. pegreffii* reach maturity and begin egg production from the 25th day of culture (Moratal et al., 2023), while it has been observed that males of *A. pegreffii* reach maturity between the 40th and 50th day (Nascetti et al., 1986; Mattiucci et al., unpublished data). In contrast, both males and females of *A. simplex* (s.s.) reach the reproductive stage around the 30th day of *in vitro* culture (Nascetti et al., 1986; Mattiucci et al., unpublished data).

The findings reported in the present study seem to suggest that some mechanisms of reproductive isolation, both *pre*- and *post-copula*, might be not fully efficient in overlapping areas. Generally speaking, hybridization events may give rise to F1 hybrids with low viability and sterility, thereby preventing gene flow between the two parental taxa. It is also possible that F1 hybrids are seldomly able to backcross with one of the two parental species, giving rise to the Fn generation of hybrids. This can result in either 50% of genome admixture in F1 hybridization or introgression (i.e. the introduction of single genes from one species into another closely related species through repeated backcrossing events). In our sample, the *Fst* values observed between the parental taxa and backcrosses at the nuclear level suggest that, despite ongoing hybridization in the contact zone between the two closely related species, some *post-copula* mechanisms of reproductive isolation may also be present, maintaining the evolutionary boundary of the species.

However, hybrid zones involve amalgamations of independently evolving genomes, and various sexual asymmetries can be disclosed by joint data from cytoplasmatic and nuclear markers. For instance, the occurrence of mito-nuclear discordance (Table 5) demonstrated by 16 specimens from the eastern Mediterranean Sea showing the nuclear genome of the parental *A. pegreffii* with the cytoplasmic genome of the parental *A. simplex* (s.s.). This mito-nuclear discordance indicates mitochondrial introgression in the genome of *A. pegreffii* in this area. In several hybrid zones, the pronounced discordance across nuclear and mitochondrial loci appears to reflect different historical appearances of introgression (Porretta and Canestrelli, 2023). Indeed, a common phenomenon is cytoplasmic ‘capture,’ where mtDNA genotypes characteristic of one species occur against the predominant nuclear background of the interacting species. The occurrence of interspecies introgression of mitochondrial genomes was detected in other parasitic nematodes, such as between *Trichinella spiralis* and *T. britovi* (Franssen et al., 2015). This phenomenon, here observed in L3-stage larvae of *A. pegreffii* from eastern Mediterranean samples at a conspicuous rate, was not previously described in other allopatric and sympatric areas of the two *Anisakis* species. However, similar findings of mito-nuclear discordance in *Anisakis* individuals – despite being based on only 1 nuclear gene locus (i.e. ITS regions of rDNA) – were reported by Mladineo et al. (2017b) from the Adriatic Sea (Croatian coast). It is important to note that in our sample only a few specimens (N = 4) displayed the most common haplotype (i.e. H7), which is shared with other populations of the parental taxon *A. simplex* (s.s.). In contrast, the remaining haplotypes (N = 12) were private, occurring only in those introgressed specimens. Among these, H18 and H19 resulted to be private haplotypes, never found in other sympatric or allopatric populations of the two parental taxa (Fig. 6; Fig. 7B), while the H20, frequently found in our samples, was previously detected from the Tunisian coast of Mediterranean Sea and from the Adriatic Sea. This pattern suggests that this finding would be the result of both past and/or paleo-introgression hybridization phenomena that occurred in the genome of the interacting species. It is hypothesized that limited hybridization between the two taxa had occurred over several episodes. Past and/or paleo-introgression phenomena could be attributed to repeated immigration of the parental taxon *A. simplex* (s.s.) from the Atlantic Ocean, which might contribute to the observed mito-nuclear discordance. Supporting this hypothesis is the finding of private haplotypes found in the Mediterranean Sea, which form a distinct mitochondrial lineage from other haplotypes (Fig. 6). These could represent remnant haplotype lineages which survived in different micro-refugia in Mediterranean waters, following a secondary contact between the two separated lineages occurred during the Plio-Pleistocene period (5.3–2.0 Ma). Similar paleo-introgression inferences have been extensively reported for other marine invertebrate taxa in the Mediterranean Sea (Schmitt et al., 2021).

Another possible explanation for this finding is that mtDNA tends to introgress more readily than nDNA, being genes contributing to reproductive isolation mechanisms generally located in the latter, rather than in mtDNA (Barton and Jones, 1983). Among additional factors driving the nuclear and cytoplasmic introgression, the asymmetry between fitness of males and females can lead to differential exchange for nuclear and cytoplasmic loci. Sex-biased processes or dissortative preference in female mate choice could play a role in mitochondrial introgression. For instance, sperm-egg recognition proteins could be generally highly specialized, making interspecific zygote formation nearly precluded between the two species. In gonochoristic parasites, it has been observed that sperms penetrating egg’s jelly coat have proteins binding specifically with receptors present on the egg of its own species (Mehlhorn, 1988). This mechanism has not been yet described in anisakid parasites, where the direct contact between females and males in the definitive host and copulation likely ensures sperm transfer. However, the asymmetry of hybrid fertility has been evoked in some animals (Mastrantonio et al., 2019) to explain instances where mtDNA appears to cross species boundaries. This may occur if fertile females leave open the possibility for interspecific cytoplasmic recombination. Hybridization between heterospecific individuals has been documented in invertebrates as playing a role even in promoting the paternal leakage and mitochondrial heteroplasmy (Mastrantonio et al., 2019), where mitochondrial genomes from both parental species can occur within an individual due to relaxing mechanisms in the egg-sperm recognition. In this context, the possible occurrence of heteroplasmy between the two *Anisakis* species, behind the mtDNA introgression, can be also considered. In theory, significant genomic divergence can be accumulated during the process of speciation, creating strong barriers to gene flow and leaving other genomic regions, such as the mitochondrial genome, more permeable to introgression (Michel et al., 2010). To clarify this phenomenon, a complete cytoplasmic genome analysis and a linkage map would also be necessary to determine the impact of any genomic rearrangements of the two species in their contact areas.

However, the maintenance of *A. pegreffii* and *A. simplex* (s.s.) as distinct phylogenetic lineages, coupled with the high level of differentiation between backcrosses and parental taxa observed in this study, despite recurrent hybridization in the contact area, suggests that certain microevolutionary processes likely act as barriers to gene flow (e.g. selection against hybrids, assortative mating and genomic incompatibility between species).

F1 hybrids may exhibit reduced viability or fertility, with these effects potentially being more pronounced in one sex. Haldane (1922) hypothesized that in hybrid populations, if one sex is absent, rare or sterile, it is typically the heterogametic sex. Consequently, ongoing introgression of nuclear genes can occur via either male or female hybrids. In our sample from the sympatric areas, 1 adult female worm was found to have an F1 genome, possessing *A. pegreffii* mtDNA (Fig. 7B), whereas the most identified F1 hybrids and backcross individuals were L3-stage larvae, possessing an *A. simplex* (s.s.) (N = 16) or *A. pegreffii* (N = 6) mtDNA (Fig. 7B). According to the SSR sex-linked loci, those 16 third-stage L3 larvae, i.e. N = 10 F1, *plus* N = 3 backcrosses, were all females, while N = 9 backcrosses were males. According to the same sex-linked loci, the sex assignment to the mitochondrial introgressed individuals resulted to be N = 3 females and N = 3 males. Thus, in our case, since most of the F1 hybrids were female worms, ongoing hybridization seems to be predominantly maintained by females. This finding seems to align with Haldane’s rule. Various causes of Haldane’s rule, such as post-zygotic sterility and inviability of hybrids, have been proposed for several vertebrates and invertebrates, including chromosomal rearrangements, incompatibility between sex chromosomes and autosomes, and the dominance theory of sex-linked genes (Cowell, 2022). These processes may contribute to maintaining reproductive boundaries between the two parasite species here studied.

Future studies on these nematode parasites, with Mendelian inheritance, will enhance our understanding of how mechanisms of reproductive isolation between closely related species drive the evolutionary force which has led to the high success of their offspring, even in sympatric and syntopic conditions. This would also explain the mechanisms that maintain species reproductive barriers, as well the relatively low frequency of hybrids (here found to be around 5%) observed in the overlapping populations of the two *Anisakis* species. Further investigation of selective pressures and patterns of genomic introgression will be necessary to determine the phenotypic traits involved in reproductive isolation between the two species, and the nature of hybridization between them.

Hybridization can also represent a fertile field for natural selection, promoting certain genotypes/haplotypes and producing more potential adaptive phenotypes with respect to the parental species in determined conditions. A ‘transgressive hybrid’ refers to hybrids expressing phenotypes which are not resident in either of the two parental taxa. This can result in the fixation of more adaptive alleles in certain genes, potentially related to new environmental variables. Indeed, since hybridization can generate both phenotypic and genetic variants, the occurrence of natural hybrids between interacting species of parasites may be linked to the presence of emerging and rapidly changing environmental factors that favour the survival of hybrid phenotypes. It could also happen that, though hybrids may be poorly adapted to either parental species’ habitats, they can sometimes outcompete parental species in changing habitats, conferring a selective advantage (King et al., 2015). In this respect, introgressive hybridization could play a key role also in the microevolutionary ecology of the *Anisakis* species in the contact zone here studied.

The current distribution of the two parental *Anisakis* species in European waters (Mattiucci et al., 2018; Díez et al., 2024) is shaped by complex biotic and abiotic factors. Water temperature and salinity play a crucial role in determining egg hatching and the survival of the first larval stages (Gomes et al., 2023). The parental genomes of the two *Anisakis* species would provide a set of genes having a selective advantage in relation to these variables, defining their temperature and salinity tolerance limits. In this context, the mixed genome of introgressed individuals may be favoured over the parental genomes at the boundaries of their distribution ranges, offering a broader temperature tolerance to their free-living-stage larvae. Interestingly, this study found that the differential pattern of hybridization along the northern Iberian Atlantic coast is mainly composed of F1 hybrids and backcrossed individuals with the parental *A. simplex* (s.s.); this area represents the northern limit of the geographical distribution of the pure parental taxon, *A. pegreffii* (Mattiucci et al., 2018; Diez et al., 2022) (Fig. 7A). The opposite trend was observed along the southern part of Atlantic Iberian coast and Alboran Sea waters, where, aside from F1 hybrids, backcrosses with the parental *A. pegreffii* were identified, mostly showing an *A. simplex* (s.s.) mitochondrial inheritance. This region represents the southern limit of the geographic distribution of the pure parental taxon *A. simplex* (s.s.) (Fig. 7A). According to this finding, we could hypothesize that the new gene combinations found in F1 and Fn individuals may enable them to tolerate marine environments that would be unfavourable to their parental species.

As the distribution of the two species of *Anisakis* in European waters may shift under climate change (Levsen et al., 2018; Diez et al., 2022; Palomba et al., 2023), eventually enlarging their contact area, the likelihood of hybridization events could increase. This could lead either to a lower fitness of the introgressed individuals following the admixture or to the enhancement of the evolutionary adaptation of the hybrid genotypes to new local conditions (King et al., 2015).

Finally, concerning the zoonotic role of these parasites, no data are so far available about the findings of hybrid genotypes identified as agents of human anisakiasis. So far, only two markers (i.e. ITS region of rDNA and/or mtDNA *cox*2) have been used to identify larval stages of etiological agents of these cases (Mattiucci et al., 2018; Palomba et al., 2023; Shamsi and Barton, 2023). Future studies, based on genotyping of larval stages causing human anisakiasis using a broader set of nuclear markers, will provide a more comprehensive genetic characterization of the larval individuals responsible for the zoonotic disease.

## Conclusions

The present work demonstrates that contemporary hybridization between the parasite taxa *A. pegreffii* and *A. simplex* (s.s.) occurs in the contact marine areas here studied, and that viable F1 hybrids have contributed to genomic introgression within the parental lineages. Since most of the hybrid individuals were identified as females based on sex-linked SSR loci, it can be hypothesized that ongoing hybridization is predominantly maintained by females in the sympatric area studied. The present study also shows the existence of *A. simplex* (s.s.) mitochondrial introgression in *A. pegreffii* from the Mediterranean Sea waters, not previously detected in other allopatric populations of the parasite species, supporting the hypothesis of paleo-introgression events occurred in these waters.

The useful application of a multilocus nuclear genotyping approach and a Bayesian analysis to investigate the genetic structure of the sister taxa included in complex species in sympatric areas, as well as to assign each individual specimen to specific hybrid categories, has been provided. Further, it has shown that the use of the only mitochondrial gene locus (i.e. mtDNA *cox*2) in the genetic/molecular identification of these parasites, especially when dealing with populations of these parasite species collected from their contact areas, can lead to the misidentification of specimens, due to the occurrence of nuclear hybridization and mitochondrial introgression phenomena.

As the distribution of the two species of *Anisakis* in European waters can shift under climate change eventually enlarging their contact area, the likelihood of hybridization events could increase. This could lead either to a lower fitness of the introgressed individuals following the admixture or to the enhancement of the evolutionary adaptation of the hybrid genotypes to new local conditions Thus, monitoring the hybridization and introgression phenomena between the two *Anisakis* species in this sympatric area would represent a tool for monitoring changes in the geographical distribution of the two species in NE Atlantic waters, under a sea temperature increase scenario.

Finally, further investigation may reveal that hybrid individuals between *A. pegreffii* and *A. simplex* (s.s.) could both alter the host-parasite co-evolutionary adaptation with their natural hosts and exhibit differential pathogenesis pathways, including those affecting accidental hosts (e.g. human infection).

## Supporting information

Mattiucci et al. supplementary material 1 Mattiucci et al. supplementary material

Mattiucci et al. supplementary material 2 Mattiucci et al. supplementary material

Mattiucci et al. supplementary material 3 Mattiucci et al. supplementary material

## Supplementary material

The supplementary material for this article can be found at https://doi.org/10.1017/S0031182025000228
