# Supplementary figures and images for: Hybridization and introgression of the mitochondrial genome between the two species Anisakis pegreffii and A. simplex (s.s.) using a wide genotyping approach: evolutionary and ecological implications
Source: Parasitology. 2025 Apr 4;152(3):293–313. doi: 10.1017/S0031182025000228 (PMC12186100; doi:10.1017/S0031182025000228)

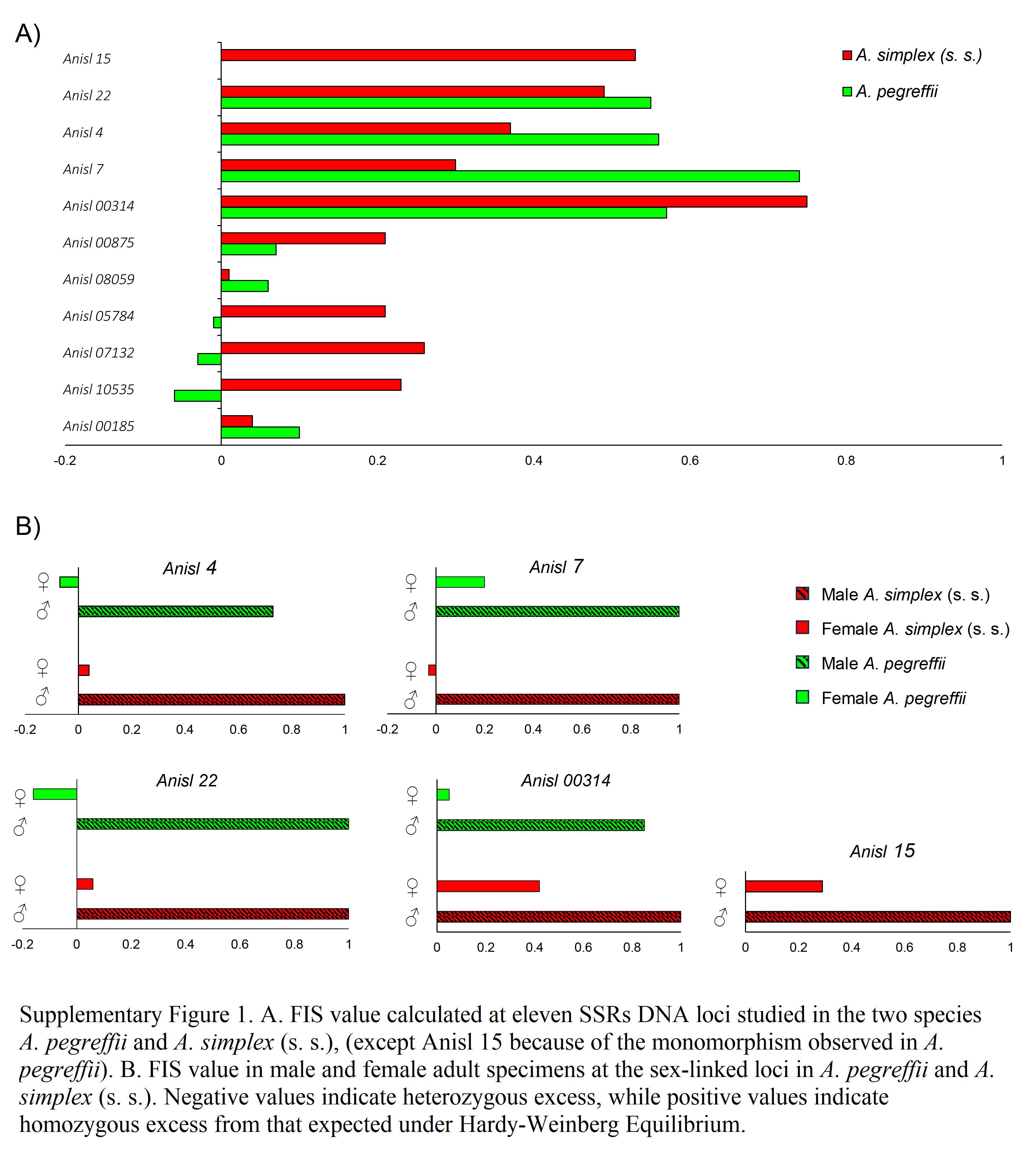

Supplement: Mattiucci et al. supplementary material 1 — Mattiucci et al. supplementary material [file S0031182025000228sup001.tif]

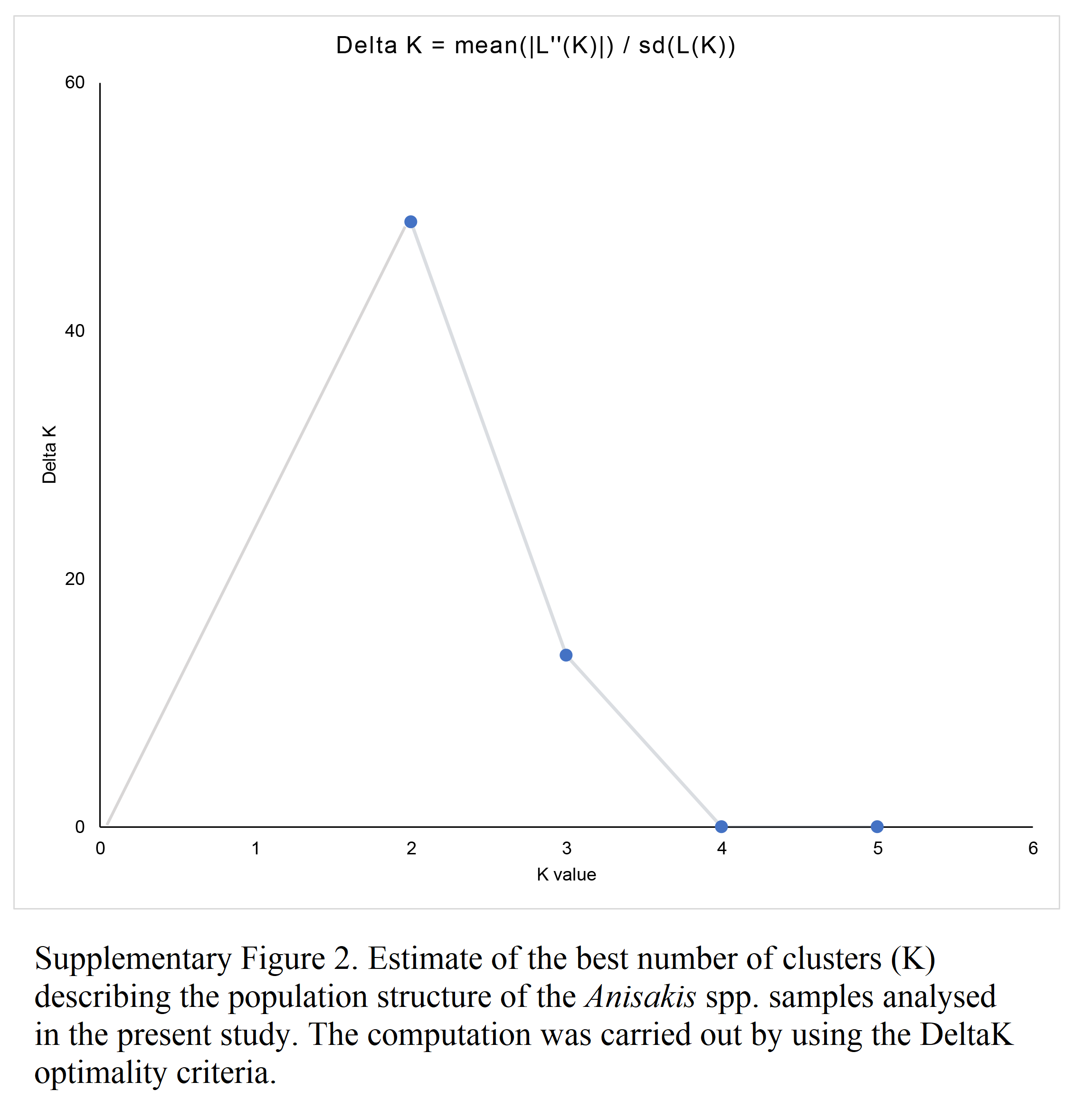

Supplement: Mattiucci et al. supplementary material 2 — Mattiucci et al. supplementary material [file S0031182025000228sup002.tif]
