# Supplementary material for: Hybridization and introgression of the mitochondrial genome between the two species Anisakis pegreffii and A. simplex (s.s.) using a wide genotyping approach: evolutionary and ecological implications
Source: Parasitology. 2025 Apr 4;152(3):293–313. doi: 10.1017/S0031182025000228 (PMC12186100; doi:10.1017/S0031182025000228)
Supplement: Mattiucci et al. supplementary material 3 — Mattiucci et al. supplementary material [file S0031182025000228sup003.doc]

**Supplementary Table 1.** Allele frequencies, observed at eleven microsatellite loci, in populations of *A. pegreffii* and *A. simplex (s. s.)* here studied. As regards the sex-linked loci (*), the most reliable estimate of allele frequencies was calculated according to the sex-linked genetic model estimate, assuming: *(i)* the hemizygosity of the males at that locus in the two *Anisakis* species; *(ii)* their adult female counterparts, as biallelic at the sex-linked loci.

| Locus | Alleles | *A. pegreffii* | *A. simplex (s. s.)* |
| --- | --- | --- | --- |
| *Anisl 00185* | *188* | 0.01 | 0.04 |
|  | *191* | 0.11 | 0.04 |
|  | *194* | 0.27 | 0.47 |
|  | *197* | 0.30 | 0.25 |
|  | *200* | 0.20 | 0.14 |
|  | *203* | 0.09 | 0.03 |
|  | *206* | 0.02 | 0.03 |
|  |  |  |  |
| *Anisl 00314* | *96* | 0.04 | 0.10 |
|  | *100* | 0.25 | 0.25 |
|  | *104* | 0.29 | 0.35 |
|  | *108* | 0.26 | 0.17 |
|  | *112* | 0.10 | 0.09 |
|  | *116* | 0.04 | - |
|  | *120* | 0.02 | 0.02 |
|  | *128* | - | 0.02 |
|  |  |  |  |
| *Anisl 10535* | *125* | - | 0.01 |
|  | *131* | 0.02 | 0.03 |
|  | *134* | 0.02 | 0.91 |
|  | *137* | 0.03 | 0.05 |
|  | *140* | 0.81 | 0.01 |
|  | *143* | 0.10 | - |
|  | *146* | 0.01 | - |
|  |  |  |  |
| *Anisl 07132* | *208* | - | 0.03 |
|  | *212* | 0.05 | 0.03 |
|  | *216* | 0.16 | 0.02 |
|  | *220* | 0.42 | 0.41 |
|  | *224* | 0.13 | 0.31 |
|  | *228* | 0.13 | 0.08 |
|  | *232* | 0.03 | 0.09 |
|  | *236* | 0.05 | 0.03 |
|  | *240* | 0.02 | - |
|  | *248* | 0.01 | - |
|  |  |  |  |
| *Anisl 05784* | *63* | - | 0.24 |
|  | *66* | - | 0.08 |
|  | *69* | - | 0.04 |
|  | *72* | - | 0.01 |
|  | *75* | - | 0.02 |
|  | *78* | 0.01 | 0.45 |
|  | *81* | 0.06 | 0.12 |
|  | *84* | 0.07 | 0.03 |
|  | *87* | 0.20 | 0.01 |
|  | *90* | 0.35 | 0.01 |
|  | *93* | 0.18 | - |
|  | *96* | 0.06 | - |
|  | *99* | 0.04 | - |
|  | *102* | 0.01 | - |
|  | *105* | - | - |
|  |  |  |  |
| *Anisl 08059* | *82* | 0.01 | 0.03 |
|  | *86* | 0.28 | 0.90 |
|  | *90* | 0.04 | 0.05 |
|  | *94* | 0.11 | - |
|  | *98* | 0.15 | 0.01 |
|  | *102* | 0.10 | 0.01 |
|  | *106* | 0.11 | - |
|  | *110* | 0.06 | - |
|  | *114* | 0.09 | - |
|  | *118* | 0.03 | - |
|  | *122* | 0.01 | - |
|  | *126* | 0.01 | - |
|  |  |  |  |
| *Anisl 00875* | *142* | - | 0.01 |
|  | *145* | - | - |
|  | *148* | 0.01 | - |
|  | *151* | 0.04 | 0.02 |
|  | *154* | 0.06 | 0.05 |
|  | *157* | 0.72 | 0.27 |
|  | *160* | 0.13 | 0.44 |
|  | *163* | 0.03 | 0.18 |
|  | *166* | - | 0.02 |
|  | *169* | - | 0.01 |
|  |  |  |  |
| *Anisl 7* | *216* | 0.16 | - |
|  | *219* | 0.50 | - |
|  | *222* | 0.31 | - |
|  | *225* | 0.03 | - |
|  | *249* | - | 0.03 |
|  | *252* | - | 0.15 |
|  | *255* | - | 0.49 |
|  | *258* | - | 0.21 |
|  | *261* | - | 0.09 |
|  | *264* | - | 0.01 |
|  | *267* | - | 0.01 |
|  | *270* | - | 0.01 |
|  |  |  |  |
| *Anisl 4* | *130* | 0.01 | - |
|  | *133* | 0.35 | - |
|  | *136* | 0.25 | - |
|  | *139* | 0.27 | - |
|  | *142* | 0.05 | - |
|  | *145* | 0.02 | - |
|  | *148* | 0.02 | - |
|  | *151* | 0.01 | - |
|  | *157* | - | 0.02 |
|  | *160* | 0.01 | 0.01 |
|  | *163* | - | 0.03 |
|  | *166* | 0.01 | 0.03 |
|  | *169* | - | 0.07 |
|  | *172* | - | 0.15 |
|  | *175* | - | 0.14 |
|  | *178* | - | 0.09 |
|  | *181* | - | 0.09 |
|  | *184* | - | 0.12 |
|  | *187* | - | 0.14 |
|  | *190* | - | 0.06 |
|  | *193* | - | 0.06 |
|  | *199* | - | 0.01 |
|  |  |  |  |
| *Anisl 22* | *182* | 0.01 | - |
|  | *185* | 0.40 | - |
|  | *188* | 0.46 | 0.01 |
|  | *191* | 0.11 | 0.02 |
|  | *194* | 0.01 | - |
|  | *200* | - | 0.10 |
|  | *203* | - | 0.02 |
|  | *206* | - | 0.02 |
|  | *209* | - | 0.03 |
|  | *212* | - | 0.06 |
|  | *215* | - | 0.06 |
|  | *218* | - | 0.18 |
|  | *221* | - | 0.04 |
|  | *224* | - | 0.08 |
|  | *227* | - | 0.06 |
|  | *230* | - | 0.05 |
|  | *233* | - | 0.05 |
|  | *236* | - | 0.07 |
|  | *239* | - | 0.02 |
|  | *242* | - | 0.01 |
|  | *245* | - | 0.05 |
|  | *248* | - | 0.04 |
|  | *251* | - | 0.01 |
|  | *260* | - | 0.02 |
|  | *263* | - | 0.01 |
|  | *266* | - | 0.01 |
|  | *272* | - | 0.01 |
|  |  |  |  |
| *Anisl 15* | *231* | 1.00 | 0.01 |
|  | *235* | - | 0.08 |
|  | *239* | - | 0.28 |
|  | *243* | - | 0.38 |
|  | *247* | - | 0.22 |
|  | *251* | - | 0.03 |
